# Entwicklung eines spindelzelligen Plattenepithelkarzinoms auf dem Boden eines lang bestehenden Pyoderma gangraenosum

**DOI:** 10.1007/s00105-022-04995-6

**Published:** 2022-04-26

**Authors:** Konstantin Drexler, Mark Berneburg, Sigrid Karrer

**Affiliations:** Klinik und Poliklinik für Dermatologie, Universitätsklinikum Regensburg, Franz-Josef-Strauß-Allee 11, 93053 Regensburg, Deutschland

**Keywords:** Plattenepithelkarzinom, Pyoderma gangraenosum, Chronische Wunde, Immunssuppression, Marjolin-Ulcus, Squamous cell carcinoma, Pyoderma gangrenosum, Chronic wound, Immunosuppression, Marjolin’s ulcer

## Abstract

Das Pyoderma gangraenosum (PG) wird den neutrophilen Dermatosen zugeordnet und präsentiert sich klinisch in Form von schmerzhaften Ulzerationen mit einem häufig livid-erythematös unterminierten Randsaum. Die Behandlung mit immunsuppressiven Medikamenten ist oft langwierig. Über die Entstehung von malignen Tumoren in einem Pyoderma gangraenosum ist bisher in der Literatur nicht berichtet worden.

## Abstract (en)

Pyoderma gangrenosum (PG) is classified as a neutrophilic dermatosis and presents clinically as painful ulcerations with undermined livid erythema at the margin. Treatment with immunosuppressive drugs is often protracted. The development of malignant tumors in pyoderma gangrenosum has not been reported in the literature.

## Anamnese

**Figure 1:**
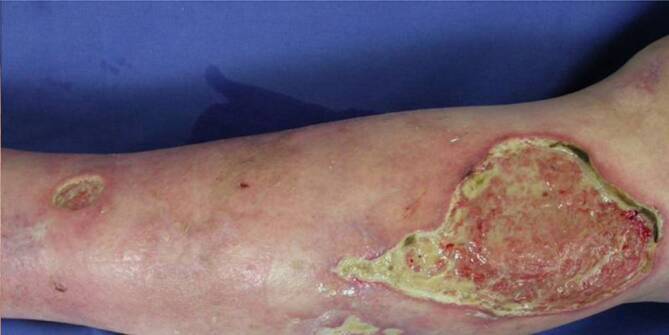

Wir berichten über einen 84-jährigen Patienten, der sich erstmalig 1999 mit mehreren seit einigen Wochen bestehenden, stark schmerzhaften Ulzerationen am linken Unterschenkel vorstellte. Klinisch zeigten sich 3 bis zu 13 cm durchmessende, scharf begrenzte Ulzerationen mit teils unterminiertem, livid-erythematösem Randsaum und fest haftenden Fibrinbelägen. Nach Ausschluss einer arteriovenösen Genese erfolgten 2 tiefe Probeexzisionen. Diese zeigten im Bereich der Dermis ein Ödem mit dichten entzündlichen Infiltraten aus Lymphozyten und zahlreichen neutrophilen Granulozyten, sodass in Zusammenschau mit der Klinik die Erstdiagnose eines PG (Pyoderma gangränosum) gestellt wurde (Abb. 1). Eine initiale intravenöse Therapie mit 80 mg/Tag Natriumprednisolon-21-succinat brachte nur eine unzureichende Besserung. Der Patient erhielt daher im weiteren Verlauf wechselnde, teils mit systemischen Glukokortikoiden kombinierte immunsuppressive Therapien mit Azathioprin, Mycophenolat-Mofetil, Infliximab, intravenösen Immunglobulinen und Cyclophosphamid. Darunter kam es zwischenzeitlich auch zur Abheilung einzelner Ulzerationen, wobei zumeist nach mechanischen Traumen immer wieder neue PG-typische Ulzera an beiden Unterschenkeln auftraten. Zwischen 2014 und 2020 stellte sich der Patient nicht mehr in unserer Klinik vor, jedoch sei es auch in diesem Zeitraum nie zu einem kompletten Stillstand der Erkrankung gekommen. Im Mai 2021 stellte sich der Patient erneut in unserer Klinik aufgrund einer Befundverschlechterung vor. Zuletzt habe er keine immunsuppressiven Medikamente mehr eingenommen.

## Befund

**Figure 2:**
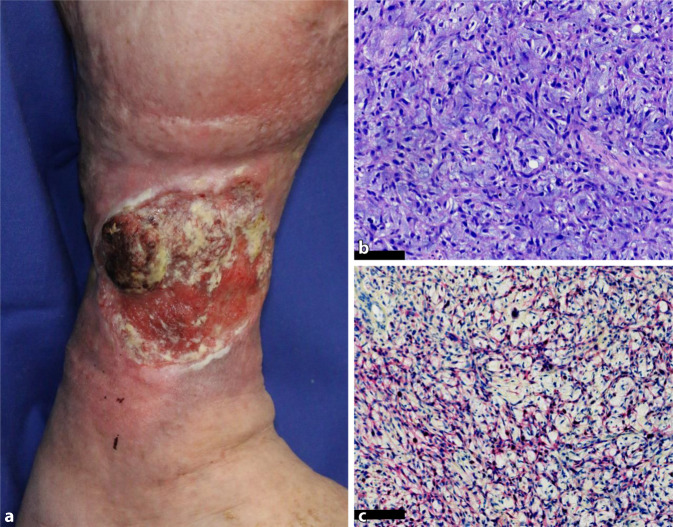

Bei der klinischen Inspektion zeigte sich am rechten Unterschenkel eine 8 × 6 cm durchmessende, fibrinbelegte Ulzeration mit deutlich livid-erythematösem Randwall. Zudem fiel ein 3,5 cm durchmessender, exophytisch wachsender, derb palpabler Nodus im Randbereich der Ulzeration auf (Abb. 2). Die genaue Bestandsdauer dieses Ulkus sowie des Tumors war anamnestisch nicht mehr zu eruieren.

## Diagnose

Eine arterielle Verschlusskrankheit und eine chronisch venöse Insuffizienz wurden erneut durch die Kollegen der Gefäßchirurgie ausgeschlossen. Es wurde der PARACELSUS-Score erhoben, der anhand von Haupt- und Nebenkriterien die Wahrscheinlichkeit für die Diagnose eines PG eingrenzt [6, 7]. Mit einem Score von 13 war bei unserem Patienten das Vorliegen eines PG sehr wahrscheinlich. Der exophytisch wachsende, ulzerierte Nodus wurde oberflächlich chirurgisch abgetragen. Histopathologisch zeigte sich das gesamte Exzidat durchsetzt von spindeligen, pleomorphen Tumorzellen mit vermehrten atypischen Mitosen. Immunhistochemisch zeigten sich die Tumorzellen positiv in der Panzytokeratin-Färbung und negativ in der Melan-A-, CK7-, CK20-, S100- und der Desmin-Färbung. Es wurde die Diagnose eines spindelzelligen, entdifferenzierten Plattenepithelkarzinoms gestellt (Abb. 2).

## Therapie und Verlauf

**Figure 3:**
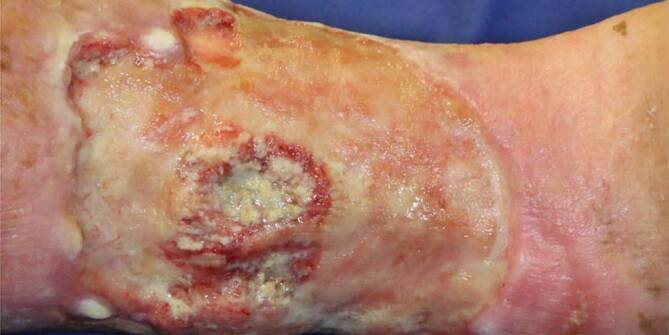

Wir leiteten zunächst nur eine topische Therapie mit Glukokortikoiden ein, worunter es zu einem leichten Abblassen der Wundränder kam. Da eine operative Sanierung des Tumors durch den Patienten nicht gewünscht war und das Risiko eines Pathergiephänomens bestand, erfolgte nach Diskussion in unserer interdisziplinären Tumorkonferenz eine Radiatio mit kumulativ 50 Gy. Darunter zeigt sich der Befund aktuell regredient (Abb. 3). In einer zusätzlich durchgeführten Staginguntersuchung mittels Computertomographie von Hals bis Becken sowie einer Magnetresonanztomographie des Schädels zeigte sich kein Hinweis auf eine Fernmetastasierung. Aktuell erfolgen regelmäßige Nachsorgeuntersuchungen. Hier zeigten sich zuletzt klinisch auch eine deutliche Verbesserung der Wundverhältnisse und kein Anhalt mehr für ein aktives Pyoderma gangraenosum.

## Diskussion

Mit einer Inzidenz von 0,63 auf 100.000 Einwohner ist das PG eine sehr seltene Erkrankung. Histopathologisch zeigen sich neutrophilenreiche entzündliche Infiltrate, die jedoch nicht spezifisch für die Erkrankung sind. Vielmehr dienen die Histologie und die klinische Untersuchung dem Ausschluss anderer ulzerierender Erkrankungen wie dem venösen oder arteriellen Ulkus, dem Ulcus hypertonicum, den Vaskulitiden, einer Kalziphylaxie oder einem malignen Tumor [1, 5]. Bei der häufig langwierigen Behandlung stehen immunsuppressive Therapien im Vordergrund. So werden in der aktuellen Leitlinie neben topischen Glukokortikoiden und Calcineurininhibitoren systemisch primär Glukokortikoide, Ciclosporin und/oder TNF-α-Inhibitoren empfohlen. Alternativ kommen auch intravenöse Immunglobuline (v. a. bei maligner Grunderkrankung), andere Immunsuppressiva wie Azathioprin, Methotrexat oder Mycophenolat-Mofetil oder andere Biologika in Betracht [2]. Von einer chirurgischen Versorgung sollte im aktiven Stadium der Erkrankung möglichst abgesehen werden, da diese zur Induktion neuer Läsionen im Sinne eines Pathergieeffektes führen kann [11]. Zu den häufigsten Komplikationen des PG gehören v. a. Wundinfektionen bis hin zur Sepsis, die auch Folge der therapiebedingten Immunsuppression sein können.

Neben einer erhöhten UV-Exposition, einer chronischen Immunsuppression, Infektionen mit humanen Papillomviren und einer Exposition gegenüber chemischen Karzinogenen stellen auch chronisch entzündliche Erkrankungen, lang bestehende Narben und chronische Wunden (sog. Marjolin-Ulkus) einen Risikofaktor für die Entwicklung eines Plattenepithelkarzinoms der Haut dar [4, 8]. Ein Marjolin-Ulkus entsteht am häufigsten auf dem Boden von Verbrennungsnarben, aber auch auf traumatisch bedingten chronischen Wunden, venösen Ulzera, Druckulzera oder einer Hidradenitis suppurativa [12]. Als Ursache für die Malignomentstehung werden in solchen Fällen eine chronische Irritation, eine wiederholte Reepithelisierung, eine lokale Schädigung der Immunabwehr der Haut, eine genetische Prädisposition oder durch lokalen Hautschaden entstandene karzinogene Toxine vermutet [12]. Die Latenz zwischen der primären Verwundung und der Manifestation des Malignoms kann zwischen 2 und 60 Jahren liegen [3]. Dabei handelt es sich meist um gut differenzierte Plattenepithelkarzinome, aber auch wenig differenzierte Subtypen und seltener auch andere Tumoren wie Basalzellkarzinome, Melanome oder Sarkome wurden berichtet [10]. Die hier dargestellte Kasuistik ist die erste, die von der Entwicklung eines Plattenepithelkarzinoms auf einem Pyoderma gangraenosum berichtet, das bei dem Patienten mit rezidivierendem Verlauf seit 22 Jahren bestand. Dies verwundert insofern nicht, da bei unserem Patienten nicht nur eine chronisch entzündliche Wunde, sondern, bedingt durch die jahrelang notwendige Therapie, auch eine Immunsuppression vorlag. Beides sind Faktoren, die das Risiko für die Entstehung eines Plattenepithelkarzinoms erhöhen [10]. Da chirurgische Eingriffe bei Patienten mit PG das Risiko einer Verschlechterung des PG mit sich bringen, stellt die Behandlung eines lokoregionären Tumors eine besondere Herausforderung dar [1]. Daher entschloss man sich bei unserem Patienten für eine Strahlentherapie. In der Behandlung des metastasierten Plattenepithelkarzinoms der Haut wird mittlerweile vermehrt der Immuncheckpoint-Inhibitor gegenüber dem „programmed cell death (PD1)“ Cemiplimab eingesetzt [8]. Hierunter wurden bereits viele verschiedene Nebenwirkungen beobachtet, unter anderem auch die Entwicklung eines PG, sodass die Indikation zur Therapie bei unserem Patienten im Falle neu auftretender Fernmetastasen kritisch diskutiert werden müsste [9].

## Fazit für die Praxis

Die Behandlung von Patienten mit PG stellt weiterhin eine Herausforderung dar, und langwierige Verläufe sind daher nicht selten. Bei lange bestehenden Ulzerationen, v. a. unter immunsuppressiver Therapie, sollten regelmäßige klinische Kontrollen erfolgen, da hier selten auch ein Plattenepithelkarzinom der Haut entstehen kann.
